# Methylation quantitative trait loci within the *TOMM20* gene are associated with metabolic syndrome-related lipid alterations in severely obese subjects

**DOI:** 10.1186/s13098-016-0171-3

**Published:** 2016-07-29

**Authors:** Juan de Toro-Martín, Frédéric Guénard, André Tchernof, Yves Deshaies, Louis Pérusse, Frédéric-Simon Hould, Stéfane Lebel, Picard Marceau, Marie-Claude Vohl

**Affiliations:** 1 Institute of Nutrition and Functional Foods (INAF), Laval University, Québec, QC Canada; 2 School of Nutrition, Laval University, Québec, QC Canada; 3 Québec Heart and Lung Institute, Québec, QC Canada; 4 Department of Medicine, Laval University, Québec, QC Canada; 5 Department of Kinesiology, Laval University, Québec, QC Canada; 6 Department of Surgery, Laval University, Québec, QC Canada

**Keywords:** *TOMM20*, Obesity, Visceral adipose tissue, meQTL, Lipid profile

## Abstract

**Background:**

The *TOMM20* gene was previously identified as differentially expressed and methylated between severely obese subjects with and without metabolic syndrome (MS). Since metabolic complications do not affect all obese patients to the same extent, the aim of this study was to identify methylation quantitative trait loci (meQTL) potentially associated with MS-related complications within the *TOMM20* locus.

**Methods:**

Methylation profiling, SNP genotyping and meQTL association tests (general linear models) were performed in a population of 48 severely obese subjects. Genotyping was extended to a larger population of 1720 severely obese subjects with or without MS, where genotype- and diplotype-based association tests were assessed by logistic regression. In silico analyses were performed using TRAP.

**Results:**

Four SNPs were identified as significant meQTLs for the differentially methylated site cg16490124. Individuals carrying rare alleles of rs4567344 (A > G) (*P* = 4.9 × 10^−2^) and rs11301 (T > C) (*P* = 5.9 × 10^−3^) showed decreased methylation levels at this site, whereas those carrying rare alleles of rs4551650 (T > C) (*P* = 3.5 × 10^−15^) and rs17523127 (C > G) (*P* = 3.5 × 10^−15^) exhibited a significant increase in methylation. rs4567344 and rs11301 were associated with increased susceptibility to exhibit high plasma triglycerides (TG ≥ 1.69 mmol/L), while rare alleles of rs4551650 and rs17523127 were significantly more represented in the low plasma total-C group (total-C ≤ 6.2 mmol/L). Haplotype reconstruction with the four meQTLs (rs4567344, rs11301, rs4551650, rs17523127) led to the identification of ten different diplotypes, with H1/H2 (GCGG/ACGG) exhibiting a nearly absence of methylation at cg16490124, and showing the highest risk of elevated plasma TG levels [OR = 2.03 (1.59–3.59)], a novel association with elevated LDL-cholesterol [OR = 1.86 (1.06–3.27)] and the complete inversion of the protective effect on total-C levels [OR = 2.03 (1.59–3.59)], especially in men. In silico analyses revealed that rs17523127 overlapped the CpG site cg16490124 and encompassed the core binding sites of the transcription factors Egr 1, 2 and 3, located within the *TOMM20* promoter region.

**Conclusion:**

This study demonstrates that *TOMM20* SNPs associated with MS-related lipid alterations are meQTLs potentially exerting their action through a CpG methylation-dependent effect. The strength of the diplotype-based associations may denote a novel meQTL additive action and point to this locus as particularly relevant in the inter-individual variability observed in the metabolic profiles of obese subjects.

**Electronic supplementary material:**

The online version of this article (doi:10.1186/s13098-016-0171-3) contains supplementary material, which is available to authorized users.

## Background

Obesity and obesity-related pathologies are major health problems worldwide and their prevalence keeps increasing, reaching epidemic proportions [1]. One of the main consequences of obesity is the development of the metabolic syndrome (MS), a cluster of metabolic disturbances, characterized by elevated waist circumference and plasma triglyceride (TG) levels, reduced HDL-cholesterol (HDL-C) levels, hyperglycemia and hypertension [2], which often progress to overt diabetes and cardiovascular diseases [3]. Among the main causes leading to the development of MS-related complications, the distribution of adipose tissue is at the foreground, with visceral adipose tissue (VAT) being considered as a key factor in the development of MS [4]. Concretely, excess VAT accumulation and dysfunction have been associated to the development of metabolic disturbances, in particular those related to lipid, glucose and amino acid metabolism [5, 6]. In this sense, an appropriate mitochondrial function is critical for lipid turnover in VAT [7] and hence adipocyte mitochondria have gained a broad interest for understanding the role of VAT in the development of MS-related complications. Importantly, adipose mitochondrial dysfunction has been found in severely obese patients, as well as in animal models of obesity and type 2 diabetes [8, 9]. These mitochondrial alterations have been linked with changes in protein levels closely related to mitochondrial biogenesis, oxidative capacity or protein import machinery [10]. On the other hand, the fact that a large proportion of obese individuals remain healthy suggests that genetic or epigenetic factors would be playing a key role in the pathogenesis of MS. To date, genetic approaches have identified a significant number of loci associated with MS, as recently reviewed [11]. Likewise, epigenetic studies have also provided potential mechanisms by which genotype and phenotype associations could be linked [12, 13]. From an integrative perspective, DNA methylation levels in so-called CpG (cytosine-phosphate-guanine) dinucleotides have been found to be partially dependent on the genetic sequence, and defined as methylation quantitative trait loci (meQTL) [14]. Previously, although not included in the final report, we identified *TOMM20* (translocase of outer mitochondrial membrane 20 homolog), a gene coding for a central component of the receptor complex involved in protein recognition and translocation to the mitochondria, as significantly overexpressed in severely obese men with vs. without MS [15]. Further studies also identified a CpG site located within the promoter region of this gene (an intergenic region shared with the *SNORA14B* locus) as significantly overmethylated in obese men with vs. without MS [13]. A recent GWAS has also found a SNP nearby the *TOMM20* promoter region as significantly associated with total-cholesterol (total-C) and LDL-cholesterol (LDL-C), which further suggests an important role of *TOMM20* in lipid metabolism [16]. Thus, to shed light on the mechanisms by which genetic variants may impact CpG methylation and how this interplay is potentially further reflected at the phenotype level, herein we integrated genetic and epigenetic approaches by identifying meQTLs located within the *TOMM20* locus for further association testing with MS-related complications.

## Methods

### Study population

The study population included 1720 patients (537 men and 1183 women) selected on the presence of severe obesity (BMI >35 kg/m^2^), who underwent biliopancreatic diversion with duodenal switch at the Quebec Heart and Lung Institute (Quebec City, Quebec, Canada). The surgical protocol and the standardized procedures to measure anthropometric and metabolic parameters are described elsewhere [17, 18]. Briefly, fasting plasma glucose levels were measured enzymatically according to the method of Richterich [19]. Total-C and TG levels were measured in plasma using enzymatic assays on a Technicon RA-500 automated analyzer (Bayer, Tarrytown, NY). Plasma HDL-C was measured in the supernatant after precipitation of LDL-C. Plasma LDL-C was estimated with the Friedewald equation [20]. The presence of MS was determined using the National Cholesterol Education Program Adult Treatment Panel III (NCEP-ATPIII) guidelines when an individual fulfilled three or more criteria [2]. All procedures were in accordance with the standards of the Laval University ethics committee and with the 1964 Helsinki declaration. Written informed consent was obtained from all individual participants included in the study.

### CpG methylation analysis

CpG methylation analysis was conducted in VAT samples of 48 severely obese (BMI >40 kg/m^2^) subjects (31 men and 17 women) selected from the study population and fulfilling at least three NCEP-ATPIII criteria. Metabolic status was taken into account to include similar proportions of individuals with and without MS (MS+ and MS−, respectively), according to NCEP-ATPIII guidelines [2]. As previously described [13], genomic DNA was extracted from 200 mg of VAT using the DNeasy Blood & Tissue kit (QIAGEN, Mississauga, Ontario, Canada), as recommended by the manufacturer. Bisulfite conversion was conducted on 1 µg of DNA, and quantitative DNA methylation analysis was carried out at the McGill University and Génome Québec Innovation Centre (Montreal, Canada), using the Infinium HumanMethylation450 BeadChip (Illumina Inc., San Diego, CA). The BeadChip interrogates more than 485,000 methylation sites at single-nucleotide resolution. Methylation data was visualized and analyzed using the GenomeStudio software version 2011.1 (Illumina Inc.) and the methylation module. Methylation levels (β values) were estimated as the ratio of signal intensity of the methylated alleles to the sum of methylated and unmethylated intensity signals of the alleles. The ratio of methylation intensities for each sample was obtained from multiple measurements of the same probe sequence (a median of 14 beads randomly distributed across the array), providing a reliable and consistent internal control probe set [21, 22]. Data correction (background subtraction and normalization) was applied using internal control probe pairs. CpG sites with a detection *P* > 0.05 were removed from analysis. β values of CpG sites located within the *TOMM20* locus (~2 kb upstream and downstream) were extracted using the GenomeStudio methylation module.

### SNP genotyping

The identification of *TOMM20* tag SNPs (tSNPs) for further genotyping was carried out using the tagger selection algorithm of the Haploview software [23, 24]. Selection of tSNPs within the *TOMM20* locus (—1:235272658-235292256, GRCh37/hg19) and flanking regions (~2 kb downstream and upstream) was performed based on linkage disequilibrium (LD r^2^ < 0.8) and minor allele frequency (MAF >5 %), considering the CEU panel (Utah residents with Northern and Western European ancestry) of the latest release of the 1000 genomes project (phase 3) [25]. Genotyping was performed on genomic DNA extracted from the blood buffy coat using the GenElute Blood Genomic DNA kit (Sigma, St. Louis, MO, USA). Selected tSNPs were genotyped in the entire population of 1720 severely obese individuals using validated primers and TaqMan probes (Applied Biosystems). Genotypes were determined using 7500 Fast Real-Time PCR System (Applied Biosystems) and analyzed using a high-throughput array technology QuantStudio 12K Flex system, coupled with Taqman OpenArray technology (Life Technologies).

### Statistical analyses

Metabolic and anthropometric differences between MS+ and MS– subjects were computed using unpaired Student’s t test. meQTL associations were tested in VAT samples using the analysis of variance (general linear model, type III sum of squares) with age, sex and BMI included in the model. Pairwise comparisons among genotype groups were performed using least square means (LS-means) when a significant meQTL was identified. Statistical significance was defined as *P* ≤ 0.05 and Benjamini-Hochberg multiple testing correction (false discovery rate [FDR]-corrected *P* ≤ 0.05) was applied. Metabolic traits were transformed to dichotomous variables and coded as low- and high-risk for a MS-related trait, according to NCEP-ATPIII criteria [Fasting glucose ≥5.6 mmol/L, total-C ≥6.2 mmol/L, LDL-C ≥4.1 mmol/L, HDL-C ≤1.03 (males) or ≤1.39 mmol/L (females), TG ≥1.69 mmol/L, Total-C/HDL-C ≥5, TAS ≥130 mmHg, TAD ≥85 mmHg]. Individuals taking any medication to treat one of these metabolic conditions were considered to meet the criteria for such condition, as previously described [26]. The Cochran–Armitage exact test for trend (two-sided) was used to compare the proportion of men and women belonging to the low- and high-risk groups for a given MS-related trait. Associations with metabolic risk groups were tested by logistic regression under a dominant model of inheritance (common homozygotes vs. heterozygotes plus rare homozygotes), adjusting the genotype effect for age, sex and BMI. The odds ratios (OR) and Wald’s confidence intervals (CI) were computed. Diplotype-based logistic models adjusted for age, sex and BMI were fitted and specific diplotype effects by sex were computed. Statistical significance was defined as *P* ≤ 0.05. Statistical analyses were performed using SAS software version 9.3 (SAS Institute, NC, USA).

### Bioinformatic analyses

Haploview software [23] was used to verify HWE and calculate MAF for each SNP. SNP imputation was performed with PLINK v1.07 [27] using the CEU panel of the 1000 Genomes Project (Phase 3) as the reference population. Haplotype reconstruction and individual diplotype assignments were inferred from genotype data using PHASE software version 2.1.1 [28] with default parameters. The predicted transcription factor (TF) binding affinities were analyzed using TRAP [29]. The sequence overlapping a differentially methylated CpG site (10 bp upstream and downstream) was used as input sequence. The Transfac vertebrates 2010.1 database was used as TF matrix and human promoter sequences were introduced as background model to compare affinities.

## Results

### Characteristics of subjects

From a total of 1720 severely obese subjects, 1717 were successfully designated either as MS+ (1402) or MS− (315). As shown in Table 1, MS+ patients showed a significant elevation of fasting glucose, waist girth and systolic blood pressure (SBP), as compared to MS− group. All plasma lipid measurements also showed significant differences between MS+ and MS− groups, with TG and total-C/HDL-C showing a significant elevation in MS+ subjects, and with total-C, LDL-C and HDL-C showing decreased levels in MS+ as compared to the MS− group. Similar significant differences were also found between MS+ and MS− subjects in the sub-population of 48 individuals used for methylation analysis. Concretely, a significant increase in fasting glucose, total-C, TG, total-C/HDL-C, SBP and DBP were found in the MS+ group, as well as a significant decrease in HDL-C levels (Table 1). On the other hand, Cochran–Armitage tests showed that men were more prone to MS than women [OR = 1.65, 95 % CI (1.27−1.84)]. In addition, a significantly higher proportion of men, as compared to women, also encompassed in the high-risk group for every MS-related trait tested, except for HDL-C (Data not shown).

**Table 1:** Characteristics of subjects

	Study population		Methylation sub-population	
	MS+	MS−	MS+	MS−
Number of subjects	1402	315	25	23
Age (years)	43.9 ± 10.5	38.7 ± 9.8***	33.9 ± 9.4	32.0 ± 7.9
BMI (kg/m^2^)	51.8 ± 8.5	51.3 ± 8.7	52.6 ± 12.7	52.3 ± 8.4
Waist girth (cm)	141.5 ± 17.2	135.6 ± 18.5***	149.4 ± 24.8	143.3 ± 17.1
Fasting glucose (mmol/L)	6.85 ± 2.43	5.11 ± 0.98***	6.61 ± 2.79	4.95 ± 0.46**
Lipid profile (mmol/L)				
Total-C	4.67 ± 0.96	4.80 ± 0.82*	4.93 ± 0.75	4.47 ± 0.87**
LDL-C	2.65 ± 0.83	2.79 ± 0.75**	2.89 ± 0.65	2.68 ± 0.77
HDL-C	1.18 ± 0.32	1.49 ± 0.37***	0.96 ± 0.19	1.28 ± 0.17***
TG	1.97 ± 1.12	1.17 ± 0.37***	2.40 ± 1.30	1.12 ± 0.45***
Total-C/HDL-C	4.18 ± 1.34	3.33 ± 0.74***	5.32 ± 1.49	3.51 ± 0.56***
Blood pressure (mm Hg)				
SBP	139 ± 16.7	134.7 ± 15.7***	145 ± 19.8	130.8 ± 13.2**
DBP	84 ± 11.3	83.0 ± 10.3	89 ± 12.0	78.0 ± 9.7**

Metabolic and anthropometric values in the study population (n = 1720) and in the methylation sub-population (n = 48) of severely obese subjects with and without MS (MS+ and MS−, respectively). Data is expressed as mean ±SD. *Asterisks* stand for significant differences between MS+ vs. MS− subjects using unpaired Student’s *t* test (*** *P* ≤ 0.0001, ** *P* ≤ 0.01, * *P* ≤ 0.05, *ns*: not significant)

*BMI* body mass index, *Total*-*C* total cholesterol, *LDL*-*C* low-density lipoprotein cholesterol, *HDL*-*C* high-density lipoprotein cholesterol, *TG* triglycerides, *SBP* systolic blood pressure, *DBP* diastolic blood pressure

### Methylation profile of *TOMM20* locus

**Fig. 1:**
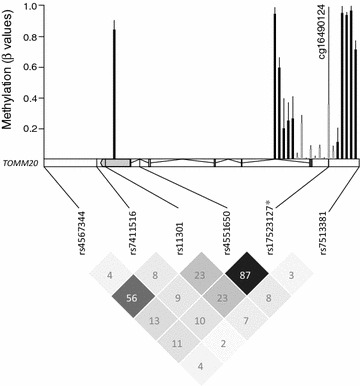
Methylation profile and linkage disequilibrium (LD) map within the *TOMM20* locus. The *upper part* of the figure shows mean methylation levels of the 20 CpG (CpG island—*grey bars*; upstream and downstream regions—*black bars*) identified within *TOMM20* locus in visceral adipose tissue samples (n = 48). Methylation levels shown as average β values ranging from 0 (unmethylated) to 1 (completely methylated). The* lower part* shows the LD map of genotyped and imputed SNPs within *TOMM20* locus in the study population (n = 1720), considering the CEU panel (Utah residents with Northern and Western European ancestry) of the latest release of the 1000 Genomes Project (Phase 3). LD r^2^ values (%) are shown in *boxes*. The imputed SNP is marked with an *asterisk*

The CpG methylation profile of *TOMM20* locus showed a total of 20 CpG sites located within a CpG island (CGI) and CGI flanking regions (Fig. 1). Mean methylation levels of these regions showed the expected distribution of methylation, with hypermethylated sites mainly located outside the CGI in both upstream and downstream CGI flanking regions (Mean β values = 0.5 and 0.7, respectively) and with CGI showing a characteristic hypomethylation (Mean β value = 0.1). We found that the CpG site cg16490124, previously reported as differentially methylated between MS+ and MS− [13], was located within the CGI and showed a large methylation heterogeneity, ranging from ~2 to ~97 % (Fig. 1).

### SNP genotyping and imputation

SNP tagging selection led to the identification of five tSNPs located downstream (rs4567344/A > G, rs7411516/G > A), at the 3′-UTR region (rs11301/T > C), at intron 4 (rs4551650/G > A) and upstream the *TOMM20* locus (rs7513381/A > G) (Fig. 1). This approach allowed us to cover 100 % of the sequence-derived genetic variability at the *TOMM20* locus. Moreover, since cg16490124 showed partial methylation, typically exhibited by CpG sites overlapping with a SNP [12], we proceeded to the imputation of previously captured SNPs during tagging selection, in order to find such a CpG-SNP [30]. SNP imputation led to the identification of a SNP (rs17523127/G > C) overlapping with cg16490124, with an imputation call rate of 99 % and a confidence threshold of 0.8. LD analysis showed high degree of LD (r^2^ = 0.87) between rs4551650 and the imputed SNP rs17523127. A certain degree of LD was also observed between rs11301 and rs4567344 (r^2^ = 0.56) (Fig. 1). All the genotyped and imputed SNPs showed a MAF >5 % and were in Hardy–Weinberg equilibrium (HWE) (Additional file 1: Table S1).

### Identification of *TOMM20* meQTL

**Fig. 2:**
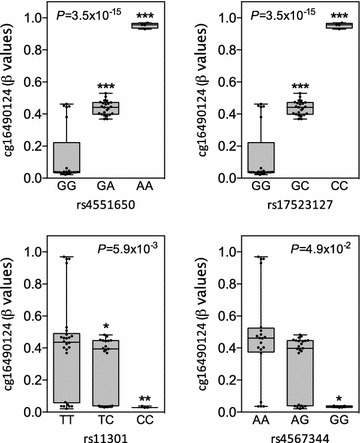
Mean degree of cg10738648 methylation for each meQTL identified in visceral adipose tissue samples. CpG methylation levels are shown as average β values ranging from 0 (unmethylated) to 1 (completely methylated). Values presented (mean ± SE) and *P* values were obtained by general linear models (type III sum of squares) adjusted for the effect of age, sex and BMI (n = 48). Pairwise comparisons among genotype groups were performed using least square means (LS-means) and *asterisks* represent significant differences vs. common homozygotes. ***P ≤ 0.0001, **P ≤ 0.01, *P ≤ 0.05

Association testing for meQTL identification was performed with genotyped and imputed SNPs in the subset of 48 severely obese subjects. As expected, rs17523127 was identified as a statistically significant meQTL for cg16490124 (*P* = 3.5 × 10^−15^). Given the strong LD between each other, rs4551650 (16.1 kb from CpG site) also showed a significant association with cg16490124 (*P* = 3.5 × 10^−15^) (Fig. 2). Methylation levels at cg16490124 were significantly higher in heterozygotes and rare homozygotes of both meQTLs than common homozygotes (Fig. 2). On the other hand, two other SNPs also showed a significant but weaker association with methylation levels of this CpG site, rs11301 (*P* = 5.9 × 10^−3^) and rs4567344 (*P* = 4.9 × 10^−2^). Interestingly, both SNPs showed the opposite relationship with cg16490124 methylation levels, i.e. rare allele carriers of rs4567344 and rs11301 (located 21.6 and 19.2 kb from CpG site, respectively) showed a significant decrease in methylation levels as compared to individuals carrying the common homozygote (Fig. 2). After Benjamini–Hochberg multiple testing correction, only rs17523127 (FDR-corrected *P* = 4.1 × 10^−13^) and rs4551650 (FDR-corrected *P* = 4.1 × 10^−13^) held a significant association with cg16490124 methylation levels.

### Association between *TOMM20* meQTLs and MS-related metabolic traits

Logistic regression analysis revealed significant associations between meQTLs and MS-related lipid traits. First, meQTLs whose rare allele had shown increased methylation levels, rs4551650 (G > A) and rs17523127 (G > C), were associated with total-C under a dominant model of inheritance. In particular, the proportion of rare allele carriers observed in the high total-C group was significantly lower than that of common homozygotes of both rs4551650 (χ^2^ = 4.8, *P* = 0.03) [OR = 0.78, 95 % CI (0.63–0.97)] and rs17523127 (χ^2^ = 4.6, *P* = 0.03) [OR = 0.78, 95 % CI (0.63–0.98)], thus suggesting a protective effect of these meQTLs for high total-C levels. This association was lost when analyzed separately in men and women (Data not shown). On the other hand, meQTLs showing a decrease in cg16490124 methylation levels, rs4567344 (A > G) and rs11301 (T > C), were significantly associated with plasma TG levels. Concretely, individuals carrying the rare allele of rs11301 (χ^2^ = 7.5, *P* = 0.006) [OR = 1.32, 95 % CI (1.08–1.62)] and rs4567344 (χ^2^ = 6.4, *P* = 0.01) [OR = 1.30, 95 % CI (1.06–1.59)] were significantly more represented in the high plasma TG group. This association was slightly higher in men for both meQTLs, rs11301 (χ^2^ = 4.6, *P* = 0.03) [OR = 1.51, 95 % CI (1.04–2.19)] and rs4567344 (χ^2^ = 6.4, *P* = 0.01) [OR = 1.64, 95 % CI (1.12–2.40)], but it was completely lost in women (Data not shown) when analyzed separately. In view that opposite associations found between meQTLs and cg16490124 methylation levels mirrored those observed with phenotype traits, we performed a diplotype-based analysis to unveil the whole effect of the simultaneous presence of these meQTLs.

### Diplotype-based phenotype associations

Haplotype reconstruction with significant meQTLs (rs4567344, rs11301, rs4551650, rs17523127) led to the identification of four major haplotypes, scattered into ten different diplotypes (Table 2). Carriers of the H2/H2 diplotypes were excluded for association analysis because of low frequency (0.7 %). The H3/H3 diplotype was considered as the reference group carrying common homozygotes of the four meQTLs. Only those diplotypes composed of common alleles of rs4551650 (G) and rs17523127 (G) and rare alleles of rs4567344 (G) and rs11301 (C), namely H1/H2 (5.1 %) and H1/H3 (18.0 %), showed significant associations with MS-related traits. In particular, H1/H2 diplotype showed the most significant associations with total-C, LDL-C and TG high-susceptibility groups in the entire population (Table 3). In view that men and women had shown a significantly different risk for MS *per se* and for most of the MS-related metabolic traits, diplotype-based analysis was performed independently in both sexes. The results obtained were remarkable in the men sub-population, where an overall OR increase in both H1/H2 and H1/H3 diplotypes was observed for total-C, LDL-C and TG high-susceptibility groups, as compared to those obtained in the entire population. By contrast, no significant differences were observed in the women sub-population (Table 3).

**Table 2:** Haplotype and diplotype patterns and frequencies for *TOMM20*

Haplotypes (H)	rs4567344 (A > G)	rs11301 (T > C)	rs4551650 (G > A)	rs17523127 (G > C)	Frequency (%)
H1	0	0	1	1	25.3
H2	1	0	1	1	10.1
H3	1	1	1	1	35.1
H4	1	1	0	0	29.5
Diplotypes					
H1/H1	0/0	0/0	1/1	1/1	6.6
H1/H2	0/1	0/0	1/1	1/1	5.1
H1/H3	0/1	0/1	1/1	1/1	18.0
H1/H4	0/1	0/1	1/0	1/0	14.4
H2/H2	1/1	0/0	1/1	1/1	0.7
H2/H3	1/1	0/1	1/1	1/1	7.2
H2/H4	1/1	0/1	1/0	1/0	6.3
H3/H3^a^	1/1	1/1	1/1	1/1	12.0
H3/H4	1/1	1/1	1/0	1/0	21.2
H4/H4	1/1	1/1	0/0	0/0	8.6

Numbers 1 and 0 indicate common and rare allele, respectively

^a^The reference diplotype

**Table 3:** Diplotype- and sex-based logistic regression analysis

		All		Men		Women	
		Wald χ^2^ (*P* value)	OR (95 % CI)	Wald χ^2^ (*P* value)	OR (95 % CI)	Wald χ^2^ (*P* value)	OR (95 % CI)
Total-C	H1/H2	5.9 (*0.01*)	2.03 (1.59–3.59)	4.7 (*0.03*)	3.00 (1.11–8.06)	1.7 (0.19)	1.60 (0.79–3–24)
	H1/H3	1.5 (0.23)	1.29 (0.85–1.96)	4.3 (*0.04*)	2.10 (1.04–4.25)	0.0 (0.99)	1.00 (0.60–1.68)
LDL–C	H1/H2	4.6 (*0.03*)	1.86 (1.06–3.27)	6.2 (*0.01*)	3.54 (1.31–9.58)	0.6 (0.45)	1.31 (0.65–2.62)
	H1/H3	1.9 (0.17)	1.34 (0.89–2.02)	7.1 (*0.008*)	2.64 (1.29–5.41)	0.1 (0.80)	0.94 (0.57–1.55)
TG	H1/H2	8.1 (*0.004*)	2.19 (1.28–3.74)	9.8 (*0.002*)	7.02 (2.07–13.76)	1.4 (0.24)	1.46 (0.78–272)
	H1/H3	3.3 (*0.03*)	1.53 (1.03–2.26)	6.1 (*0.01*)	2.43 (1.20–4.93)	0.3 (0.57)	1.14 (0.73–1.78)

Diplotype-based logistic regression analyses were fitted including sex, age and BMI as confounding factors in the entire population (n = 1720), and including age and BMI when men and women were analyzed separately (n = 537 and n = 1183, respectively). The H3/H3 diplotype was set as the reference group for comparison purposes since it carries wild type alleles of the four meQTLs tested. Significant Wald-tests are shown in italic face (*P* ≤ 0.05)

*OR* odds ratio, *CI* 95 % Wald’s confidence intervals, *Total*-*C* total cholesterol, *LDL*-*C* low-density lipoprotein cholesterol, *TG* triglycerides

### Diplotype-based meQTL associations and impact on TF binding affinity

**Fig. 3:**
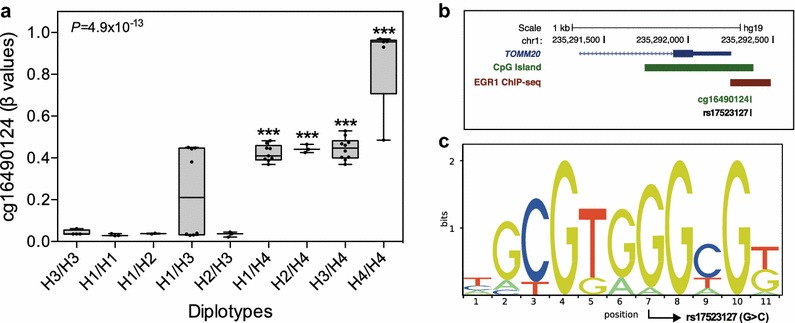
Mean methylation levels of cg10738648 for each diplotype and a schematic representation of Erg1-binding sites. **a** Mean degree of cg16490124 methylation levels for diplotypes in visceral adipose tissue samples. CpG methylation levels are shown as average β values ranging from 0 (unmethylated) to 1 (completely methylated). Values presented (mean ±SE) and *P* values were obtained by general linear models (type III sum of squares) adjusted for the effect of age, sex and BMI (n = 48). *Asterisks* represent significant differences vs. H3/H3 (reference diplotype). ***P ≤ 0.0001. **b** Schematic representation of the *TOMM20* promoter region showing Erg1-binding sites (*horizontal red bar*), CpG island (*horizontal green bar*), cg16490124 and rs175231127, obtained from ENCODE data in the UCSC genome browser. **c** Sequence logo is the graphical representation of the Egr1 position-specific scoring matrix (PSSM) identified by TRAP and obtained from the Transfac database (ID: M00243). Sequence logo shows the base preference, sized and sorted relative to their occurrence in the PSSM. The location of the CpG-SNP rs175231127 (G > C) within the Egr1-binding site is marked with an *arrow*

Diplotype-based logistic models suggested that associations found between diplotypes H1/H2 and H1/H3 with MS-related lipid traits could be due to the absence of methylation at cg16490124. meQTL association test revealed that H1/H2, the diplotype showing the strongest associations with total-C, LDL-C and TG levels, exhibited a nearly absence of methylation at cg16490124 (Fig. 3a). On the other hand, H1/H3 showed partial methylation and, as expected, those diplotypes carrying rare homozygotes of both rs4551650 (AA) and rs17523127 (CC) showed higher methylation levels (Fig. 3a). Since the association between meQTL and lipid profile appeared to be modulated by methylation levels at cg16490124, located within the promoter region of *TOMM20*, we investigated whether this CpG site was encompassed by TF binding sites (TFBS). On the inclusion of a flanking sequence of 10 bp upstream and downstream the CpG site, TRAP results revealed that cg16490124 encompassed several putative TFBS, according to Transfac matrix similarities (Data not shown). Interestingly, the most significantly overrepresented TF binding matrices, that remained significant after multiple testing correction, corresponded to Early growth response proteins 1, 2 and 3 [Egr1 (*P* = 6.3 × 10^−05^), Egr2 (*P* = 1.7 × 10^−07^) and Egr3 (*P* = 3.6 × 10^−06^)], three members of the Egr family of zinc finger TFs. Position-specific scoring matrices showed identical consensus sequence at the TFBS core region of the three Egr (Additional file 1: Figure S1). Using the ENCODE ChIP-Seq data in UCSC genome browser, we discovered a peak of Egr1-binding at *TOMM20* promoter region, overlapping with cg16490124 (Fig. 3b). Finally, the comparison of the input sequence used for TRAP prediction with Egr1-binding sites, led to the identification of the CpG-SNP rs17523127 as being located within the TFBS core region (Fig. 3c).

## Discussion

In the present work, we aimed to analyze whether potential meQTLs located within the *TOMM20* locus were associated with MS-related metabolic traits. In this regard, recent studies focused on the interplay between CpG methylation levels and the underlying genetic sequence, as carried out here, have emerged as suitable tools to detect disease biomarkers [34–36]. Overall, the main findings of this study reinforce the assumption that the link between epigenetics and the underlying genetic sequence may be further reflected in the phenotype, as previously highlighted [33]. In particular, significant meQTLs identified within the *TOMM20* locus showed relevant associations with MS-related lipid traits, specifically with higher susceptibility of increased total-C, LDL-C and TG levels. Interestingly, two of the meQTLs identified within the *TOMM20* locus, rs11301 and rs4567344, whose rare alleles showed a decrease in methylation levels of cg16490124, were both significantly associated with increased risk of high TG levels. By contrast, individuals carrying rare alleles of rs4551650 and rs17523127 showed increased methylation levels at the same CpG site and a protective effect on total-C levels. Since the impact of meQTL on phenotype traits are eventually due to an additive effect of two or more genetic variants, leading to a sum of small effects [31], we hypothesized that relatively weak associations obtained with independent genotypes could be largely explained by a diplotype effect. As such, H1/H2 and H1/H3 diplotypes were more strongly associated with increased susceptibility to exhibit high TG levels than the single genotypes (rs11301 and rs4567344), suggesting a potential additive effect of both meQTLs. Moreover, diplotype analyses provide a more accurate and powerful estimation of the genetic effects on multifactorial-disease traits, and might unveil a larger overall effect if antagonistic genetic interactions are present [32]. Intriguingly, we provide evidence that diplotypes composed of rare allele carriers from rs11301 and rs4567344 and common homozygotes from rs4551650 and rs17523127 not only abolished the protective effect shown independently by rs4551650 and rs17523127 on total-C levels, but they were in fact significantly associated with a high risk of exhibiting elevated total-C and LDL-C levels, thus suggesting a CpG methylation-mediated effect. In this sense, non-coding SNPs as those tested here are supposed to have an impact on the phenotype through indirect means, e.g. altering methylation levels (meQTL) or TFBS [33], which does not necessarily imply a linear correlation with a quantitative gene expression change (eQTL) or a phenotype trait [34], but an additive association model. Then, since polymorphisms tested in complex diseases rarely account for a large proportion of the variance, it was thus unlikely to obtain high significance levels unless a very impressive cohort was used. In this context, corrections for multiple testing would lead to too stringent significance threshold and may result in false negatives in such a discovery study. Instead, the potential associations with phenotype traits were assessed by logistic regression adjusted for age, sex and BMI to avoid the occurrence of casual relationships, and subsequently tested by means of a diplotype-based logistic regression, in order to detect stronger associations supporting previous results. Indeed, as previously mentioned, hidden associations revealed by diplotype-based analysis suggested that methylation levels at cg16490124 could be modulating the impact of SNPs on the phenotype. In this sense, one of the functional outcomes derived from altered gene methylation profile at promoter regions is usually the alteration of TFBS.

The transcriptomic study in which *TOMM20* showed a significantly overexpression in subjects with MS was performed in a relatively small cohort of 14 severely obese men [15], which made not possible to conduct association studies or correlation analysis with methylation levels with sufficient statistical power. Nonetheless, the goal of this study was not specifically to find out this functional outcome, but to focus on the potential association between *TOMM20* SNPs and obesity-associated metabolic traits. In any case, we did study the potential effect of the differentially methylated CpG site cg16490124 on *TOMM20* transcription regulation by analyzing its impact at TFBS. It is worth noting that cg16490124 is located within a typically hypomethylated CpG island which mainly encompasses the binding sites for nuclear respiratory factor 2 (Nrf-2), previously described as a required TF for *TOMM20* gene expression [35, 36]. Intriguingly, when the CpG surrounding sequence was examined for TF binding affinities, Nrf-2 binding site was not found among the most significant motifs. By contrast, highly significant affinities were found for Egr1-, Egr2- and Egr3-binding sites. Egr1 and Egr2 proteins are closely related to adipocyte differentiation, with Egr2 pro-adipogenic effects being potentiated by Egr1 knockdown [37]. Moreover, a strong correlation between Egr1 expression levels with dietary-induced obesity has been found in both mice and humans [38]. In this context, Egr-1 null mice would be protected against obesity-related complications, such as elevated circulating TG levels, by a mechanism possibly mediated by FOXC2, increasing energy expenditure and altering mitochondrial function [38]. Since *TOMM20* has a key role in maintaining mitochondrial biogenesis and function [36, 39, 40], the significant affinities found between Egr proteins with the underlying sequence of cg16490124 point to a potential new pathway leading to adipocyte dysfunction. Thus, it is conceivable that increased methylation levels of cg16490124, located within *TOMM20* promoter and encompassed by Egr-binding sites, results in decreased Egr1 binding, thus leading to a protective effect as that observed between rs4551650 and rs17523127 with total-C levels. Therefore, a potential alteration on these TFBS could account for the observed alterations of MS-related lipid features in severely obese patients. Although we do acknowledge that in silico studies do not represent a mechanistic cause of the disease and need to be validated by functional studies, we consider that these results pointing to Egr proteins agree with *TOMM20* physiological function and suggest a potential role of this gene in the development of dysfunctional adipocytes. These results thus represent a starting point for further functional studies focused on assessing the actual impact of meQTLs using functional genomics approaches, including transfection assays or measurements of gene expression in cells derived from carriers/non-carriers of the above mentioned meQTLs.

Finally, a stronger risk of high total-C, LDL-C and TG levels was found in men carrying H1/H2 and H1/H3 diplotypes, as compared to women. Although it has been well established that energy expenditure and lipid homeostasis differ between men and women [41], the underlying mechanisms leading to these sex differences remain to be elucidated. Herein, we have noted that men were more prone than women to MS and to most of MS-related complications. Regarding MS-related lipid traits, the impact of meQTLs on the phenotype mirrored these differences, thus providing a clue to understand the different susceptibility to MS between men and women, as previously suggested by others [42–44].

## Conclusions

This study demonstrates that *TOMM20* SNPs associated with MS-related lipid alterations are meQTLs thus potentially exerting their action in a CpG methylation-dependent way. The strength of the diplotype-based associations with total-C, LDL-C and TG levels may denote a novel meQTL additive action, an effect worthy of further analysis in larger methylation cohorts to broaden the knowledge of interactions between gene variation and CpG methylation on the susceptibility to MS-related complications.

## Additional file

10.1186/s13098-016-0171-3 Genotype distribution of genotyped and imputed SNPs within the TOMM20 locus. **Figure S1.** Sequence logos are the graphical representation of the Egr1 (a), Egr2 (b) and Egr3 (c) position-specific scoring matrix (PSSM) identified by TRAP and obtained from the Transfac database (IDs: M00243, M00245 and M00246, respectively). Sequence logo shows the base preference, sized and sorted relative to their occurrence in the PSSM.
