# Supplementary material for: Methylation quantitative trait loci within the TOMM20 gene are associated with metabolic syndrome-related lipid alterations in severely obese subjects
Source: Diabetol Metab Syndr. 2016 Jul 29;8:55. doi: 10.1186/s13098-016-0171-3 (PMC4966599; doi:10.1186/s13098-016-0171-3)
Supplement: Supplementary file 1 — 10.1186/s13098-016-0171-3 Genotype distribution of genotyped and imputed SNPs within the TOMM20 locus. Figure S1. Sequence logos are the graphical representation of the Egr1 (a), Egr2 (b) and Egr3 (c) position-specific scoring matrix (PSSM) identified by TRAP and obtained from the Transfac database (IDs: M00243, M00245 and M00246, respectively). Sequence logo shows the base preference, sized and sorted relative to their occurrence in the PSSM. [file 13098_2016_171_MOESM1_ESM.docx]

Methylation quantitative trait loci within the *TOMM20* gene are associated with metabolic syndrome-related lipid alterations in severely obese subjects

Juan de Toro-Martín^a,b^, Frédéric Guénard^a,b^, André Tchernof^b,c^, Yves Deshaies^c,d^, Louis Pérusse^a,e^, Frédéric-Simon Hould^f^, Stéfane Lebel^f^, Picard Marceau^f^, and Marie-Claude Vohl^a,b,*^

**^a^** Institute of Nutrition and Functional Foods (INAF), Laval University, Québec, QC, Canada;

**^b^** School of Nutrition, Laval University, Québec, QC, Canada;

**^c^** Québec Heart and Lung Institute, Québec, QC, Canada;

**^d^** Department of Medicine, Laval University, Québec, QC, Canada;

**^e^** Department of Kinesiology, Laval University, Québec, QC, Canada;

**^f^** Department of Surgery, Laval University, Québec, QC, Canada.

***Corresponding author**:

marie-claude.vohl@fsaa.ulaval.ca;

Tel.: +1 (418) 656-2131 (ext. 4676);

Fax: +1 (418) 656-5877.

**Supplementary Material**

**Table S1.** Genotype distribution of genotyped and imputed SNPs within the *TOMM20* locus.

| rs number | Localization | Region relative to gene | Number of genotypes | Common HMZ | HTZ | Rare HMZ | MAF | HWE  *P* value |
| --- | --- | --- | --- | --- | --- | --- | --- | --- |
|  |  |  |  |  |  |  |  |  |
| rs4567344 | g.235270774A>G | Downstream | 1732 | 970 | 648 | 114 | 0.25 | 0.68 |
| rs7411516 | c.2723+299G>A | Downstream | 1733 | 1189 | 495 | 49 | 0.17 | 0.77 |
| rs11301 | c.2200T>C | 3'-UTR | 1728 | 717 | 797 | 214 | 0.35 | 0.74 |
| rs4551650 | c.393+794G>A | Intron | 1727 | 854 | 721 | 152 | 0.30 | 0.99 |
| rs17523127* | c.-339G>C | Upstream | 1709 | 847 | 712 | 150 | 0.30 | 0.98 |
| rs7513381 | c.-2085A>G | Upstream | 1736 | 1322 | 388 | 26 | 0.13 | 0.68 |

SNP localization is relative to NM_014765.2 coding sequence (c.) and to NC_000001.10 genomic sequence (g) from the GRCh37/hg19 reference genome. HMZ: homozygotes, HTZ: heterozygotes, MAF: minor allele frequency, HWE: Hardy-Weinberg equilibrium, UTR: untranslated region. The asterisk represents the imputed SNP.

**Figure S1.** Sequence logos are the graphical representation of the Egr1 (a), Egr2 (b) and Egr3 (c) position-specific scoring matrix (PSSM) identified by TRAP and obtained from the Transfac database (IDs: M00243, M00245 and M00246, respectively). Sequence logo shows the base preference, sized and sorted relative to their occurrence in the PSSM.
